# Ethyl 2,6-bis­(4-bromo­phen­yl)-1-iso­cyano-4-oxo­cyclo­hexa­necarboxyl­ate

**DOI:** 10.1107/S160053681401530X

**Published:** 2014-07-05

**Authors:** Dawei Zhang, Linlin Hao, Jing Li

**Affiliations:** a College of Agricultural Sciences, Jilin University, Changchun, Jilin Province 130062, People’s Republic of China; b College of Life Sciences and Biotechnology, Heilongjiang Bayi Agricultural University, Heilongjiang Province 163319, People’s Republic of China

**Keywords:** crystal structure

## Abstract

In the title compound, C_22_H_19_Br_2_NO_3_, the central oxo­cyclo­hexane ring is in a twist-boat conformation; all the substituents (one eth­oxy­carbonyl and two aryl groups) are located in equatorial orientations. One of the –CH_2_– groups and the opposite –CH– group bearing a bromo­benzene substituent form the flagpoles of the twist-boat. The dihedral angle between the aromatic rings is 76.4 (4)°. In the crystal, weak C—H⋯O inter­actions link the mol­ecules into *C*(5) chains propagating in the [010] direction. A short Br⋯O contact of 3.254 (4) Å is observed.

## Related literature   

For further details of the synthesis, see: Tan *et al.* (2009 ▶); Zhang *et al.* (2010 ▶). For more [5 + 1] annulation reactions, see: Bi *et al.* (2005 ▶); Dong *et al.* (2005 ▶); Hu *et al.* (2008 ▶); Zhao *et al.* (2006 ▶); Fu *et al.* (2009 ▶); Xu *et al.* (2012 ▶).
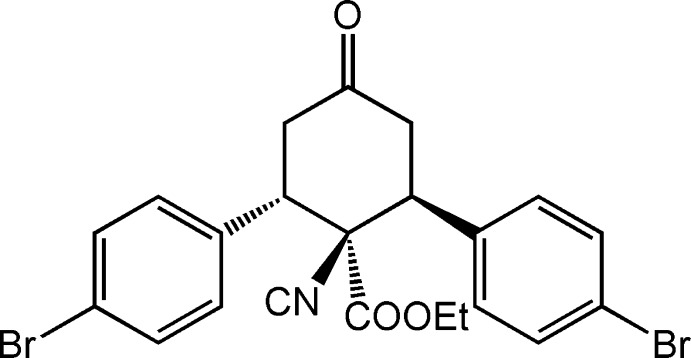


## Experimental   

### 

#### Crystal data  

C_22_H_19_Br_2_NO_3_
*M* *_r_* = 505.20
Monoclinic, 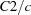

*a* = 21.9920 (17) Å
*b* = 11.0750 (19) Å
*c* = 17.648 (3) Å
β = 103.560 (2)°
*V* = 4178.6 (11) Å^3^
*Z* = 8
Mo *K*α radiation
μ = 3.90 mm^−1^
*T* = 293 K
0.17 × 0.16 × 0.13 mm

#### Data collection  

Bruker SMART APEXII CCD diffractometer
Absorption correction: multi-scan (*SADABS*; Bruker, 2007 ▶) *T*_min_ = 0.557, *T*_max_ = 0.631
10763 measured reflections
3904 independent reflections
2578 reflections with *I* > 2σ(*I*)
*R*_int_ = 0.026

#### Refinement  

*R*[*F* ^2^ > 2σ(*F* ^2^)] = 0.043
*wR*(*F* ^2^) = 0.113
*S* = 1.03
3904 reflections
253 parameters
H-atom parameters constrained
Δρ_max_ = 1.11 e Å^−3^
Δρ_min_ = −0.92 e Å^−3^

### 

Data collection: *APEX2* (Bruker, 2007 ▶); cell refinement: *SAINT* (Bruker, 2007 ▶); data reduction: *SAINT*; program(s) used to solve structure: *SHELXS97* (Sheldrick, 2008 ▶); program(s) used to refine structure: *SHELXL97* (Sheldrick, 2008 ▶); molecular graphics: *SHELXTL* (Sheldrick, 2008 ▶); software used to prepare material for publication: *SHELXTL*.

## Supplementary Material

Crystal structure: contains datablock(s) I. DOI: 10.1107/S160053681401530X/hb7238sup1.cif

Structure factors: contains datablock(s) I. DOI: 10.1107/S160053681401530X/hb7238Isup2.hkl

Supporting information file. DOI: 10.1107/S160053681401530X/hb7238Isup3.cml

CCDC reference: 1011009

Additional supporting information:  crystallographic information; 3D view; checkCIF report

## Figures and Tables

**Table 1:** Hydrogen-bond geometry (Å, °)

*D*—H⋯*A*	*D*—H	H⋯*A*	*D*⋯*A*	*D*—H⋯*A*
C11—H11⋯O3^i^	0.98	2.58	3.226 (5)	123

Symmetry code: (i) 
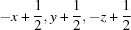
.

**Fig. 1.**
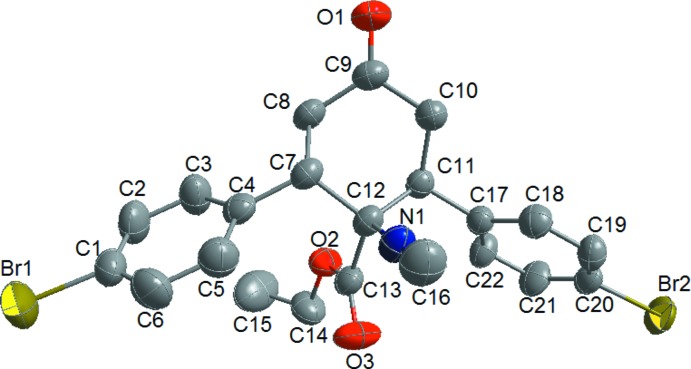
View of the molecular structure of the title compound with displacement ellipsoids drawn at the 30% probability level.

## supplementary crystallographic information

### S2.1. Synthesis and crystallization

To a mixture of (1E,4E)-1,5-bis­(4-bromo­phenyl)­penta-1,4-dien-3-one (392 mg, 1.0 mmol) and ethyl iso­cyano­acetate (0.132 mL, 1.2 mmol) in DMF (5 ml) was added 1,8-di­aza­bicyclo­[5.4.0]undec-7-ene (DBU) (0.015 mL, 0.1 mmol) in one portion at room temperature. The reaction mixture was stirred at room temperature, and the reaction mixture was monitored by TLC. After the substrate (1E, 4E)-1,5-bis­(4-bromo­phenyl)­penta-1,4-dien-3-one was consumed, the resulting mixture was poured into ice-water (30 ml) under stirring. The precipitated solid was collected by filtration, washed with water (3 × 10 ml), and dried under vacuum to afford the crude product which was purified by flash chromatography (silica gel, petroleum ether : di­ethyl ether = 3:1, v/v) to give the title compound (460 mg, 91%). The material was recrystallized from a mixture of petroleum ether and di­ethyl ether to provide colourless blocks. For further synthesis details, see: Tan *et al.* (2009); Zhang *et al.* (2010).

### S2.2. Refinement

Crystal data, data collection and structure refinement details are summarized in Table 1.

Hydrogen atoms were generated in idealized positions (according to the *sp*2 or *sp*3 geometries of their parent carbon), and then refined using a riding model with fixed C—H distances (C—H = 0.95–1.00 Å) and with *U*iso(H) = 1.2*U*eq(C).

### S3. Results and discussion

Comment

In the process of strategies developing of [5+1] annulation for the construction of six-membered cyclic compounds, we have found that ethyl iso­cyano­acetate is an active carbon nucleophile that can react with di­vinyl ketone through a tandem double Michael-addition cyclization. This one-step annulation can regiospecificly forms highly constrained cyclo­hexane analogues of phenyl­analine (Phe) which are precursors for the synthesis of peptide analogues with controlled fold in the backbone. The constrained ring systems play important roles in restricting torsional angle χ1 and in peptide receptor recognition processes, thus the [5+1] annulation reactions have drew much attentions and both the five-carbon 1,5-bielectrophiles and the one-atom nucleophiles been explored extensively (Bi *et al.*, 2005; Dong *et al.*, 2005; Hu *et al.*, 2008; Zhao *et al.*, 2006; Fu *et al.*, 2009; Xu *et al.*, 2012).

The title compound, a phenyl substituted highly constrained cyclo­hexane analogue of Phe, is one of the products obtained during the study of [5+1] annulation of di­vinyl ketone and iso­cyano­acetate. In the crystal, the central six-member oxo­cyclo­hexane ring adopts a twist-boat conformation (Fig. 1), and all of the ethoxyl carbonyl and two aryl groups are located in equatorial positions. The aryl groups are *trans* to each other and the dihedral angle between two aromatic rings is 76.45 (4) °. In this molecular, C11 with axial hydrogen and C8 (CH_2_) are on the flagpole positions of the boat conformation, which give the least torsional strain. C12 and C7 are on one side of the boat conformation, and their equatorial substituents, ethoxyl carbonyl and aryl groups, fit in with the formation boat conformation of this compound.

### Crystal data

**Table:**

C_22_H_19_Br_2_NO_3_	*F*(000) = 2016
*M**_r_* = 505.20	*D*_x_ = 1.606 Mg m^−^^3^
Monoclinic, *C*2/*c*	Mo *K*α radiation, λ = 0.71069 Å
Hall symbol: -C 2yc	Cell parameters from 78 reflections
*a* = 21.9920 (17) Å	θ = 1.3–26.0°
*b* = 11.0750 (19) Å	µ = 3.90 mm^−^^1^
*c* = 17.648 (3) Å	*T* = 293 K
β = 103.560 (2)°	BLOCK, colorless
*V* = 4178.6 (11) Å^3^	0.17 × 0.16 × 0.13 mm
*Z* = 8	

### Data collection

**Table:**

Bruker SMART APEXII CCD diffractometer	3904 independent reflections
Radiation source: fine-focus sealed tube	2578 reflections with *I* > 2σ(*I*)
Graphite monochromator	*R*_int_ = 0.026
ω scans	θ_max_ = 25.6°, θ_min_ = 1.9°
Absorption correction: multi-scan (*SADABS*; Bruker, 2007)	*h* = −26→25
*T*_min_ = 0.557, *T*_max_ = 0.631	*k* = −13→13
10763 measured reflections	*l* = −21→18

### Refinement

**Table:**

Refinement on *F*^2^	Primary atom site location: structure-invariant direct methods
Least-squares matrix: full	Secondary atom site location: difference Fourier map
*R*[*F*^2^ > 2σ(*F*^2^)] = 0.043	Hydrogen site location: inferred from neighbouring sites
*wR*(*F*^2^) = 0.113	H-atom parameters constrained
*S* = 1.03	*w* = 1/[σ^2^(*F*_o_^2^) + (0.0466*P*)^2^ + 7.9699*P*] where *P* = (*F*_o_^2^ + 2*F*_c_^2^)/3
3904 reflections	(Δ/σ)_max_ = 0.001
253 parameters	Δρ_max_ = 1.11 e Å^−^^3^
0 restraints	Δρ_min_ = −0.92 e Å^−^^3^

### Special details

**Table:**

Geometry. All e.s.d.'s (except the e.s.d. in the dihedral angle between two l.s. planes) are estimated using the full covariance matrix. The cell e.s.d.'s are taken into account individually in the estimation of e.s.d.'s in distances, angles and torsion angles; correlations between e.s.d.'s in cell parameters are only used when they are defined by crystal symmetry. An approximate (isotropic) treatment of cell e.s.d.'s is used for estimating e.s.d.'s involving l.s. planes.
Refinement. Refinement of *F*^2^ against ALL reflections. The weighted *R*-factor *wR* and goodness of fit *S* are based on *F*^2^, conventional *R*-factors *R* are based on *F*, with *F* set to zero for negative *F*^2^. The threshold expression of *F*^2^ > σ(*F*^2^) is used only for calculating *R*-factors(gt) *etc*. and is not relevant to the choice of reflections for refinement. *R*-factors based on *F*^2^ are statistically about twice as large as those based on *F*, and *R*– factors based on ALL data will be even larger.

### Fractional atomic coordinates and isotropic or equivalent isotropic displacement parameters (Å^2^)

**Table:**

	*x*	*y*	*z*	*U*_iso_*/*U*_eq_	
Br2	−0.01743 (2)	0.57808 (4)	0.12001 (3)	0.06681 (18)	
Br1	0.59543 (3)	0.56713 (6)	0.49860 (4)	0.0979 (2)	
O2	0.30729 (12)	0.7231 (2)	0.34697 (15)	0.0512 (7)	
C17	0.19183 (17)	0.6993 (3)	0.1672 (2)	0.0406 (9)	
C11	0.25738 (17)	0.7509 (3)	0.1820 (2)	0.0401 (8)	
H11	0.2588	0.8177	0.2188	0.048*	
N1	0.30420 (15)	0.5508 (3)	0.1772 (2)	0.0505 (8)	
C10	0.27366 (18)	0.8048 (4)	0.1094 (2)	0.0468 (9)	
H10A	0.2798	0.7402	0.0749	0.056*	
H10B	0.2392	0.8544	0.0818	0.056*	
C12	0.30963 (17)	0.6605 (3)	0.2217 (2)	0.0409 (9)	
O3	0.28934 (16)	0.5274 (3)	0.32118 (18)	0.0754 (10)	
C13	0.30092 (17)	0.6265 (4)	0.3028 (2)	0.0453 (9)	
C9	0.33168 (19)	0.8799 (4)	0.1308 (2)	0.0523 (10)	
C7	0.37673 (17)	0.7151 (3)	0.2243 (2)	0.0443 (9)	
H7	0.3893	0.6799	0.1794	0.053*	
O1	0.34440 (16)	0.9555 (3)	0.08779 (19)	0.0795 (10)	
C18	0.16493 (18)	0.6331 (4)	0.1019 (2)	0.0495 (10)	
H18	0.1885	0.6140	0.0661	0.059*	
C22	0.15553 (18)	0.7240 (4)	0.2200 (2)	0.0521 (10)	
H22	0.1731	0.7666	0.2653	0.062*	
C4	0.42738 (17)	0.6782 (4)	0.2946 (2)	0.0470 (9)	
C5	0.4465 (2)	0.5596 (4)	0.3050 (3)	0.0652 (12)	
H5	0.4261	0.5013	0.2703	0.078*	
C1	0.5261 (2)	0.6100 (5)	0.4171 (3)	0.0622 (12)	
C19	0.10326 (19)	0.5942 (4)	0.0883 (2)	0.0512 (10)	
H19	0.0859	0.5486	0.0443	0.061*	
C20	0.06841 (17)	0.6234 (3)	0.1403 (2)	0.0475 (9)	
C14	0.2992 (2)	0.7071 (5)	0.4265 (2)	0.0679 (13)	
H14A	0.2574	0.7320	0.4290	0.082*	
H14B	0.3043	0.6226	0.4411	0.082*	
C8	0.37386 (18)	0.8521 (4)	0.2093 (2)	0.0518 (10)	
H8A	0.3582	0.8925	0.2497	0.062*	
H8B	0.4156	0.8824	0.2112	0.062*	
C21	0.09395 (19)	0.6872 (4)	0.2070 (3)	0.0584 (11)	
H21	0.0702	0.7052	0.2428	0.070*	
C2	0.5071 (2)	0.7263 (5)	0.4093 (3)	0.0709 (13)	
H2	0.5272	0.7839	0.4448	0.085*	
C3	0.4580 (2)	0.7598 (4)	0.3488 (3)	0.0653 (12)	
H3	0.4453	0.8401	0.3446	0.078*	
C16	0.3003 (3)	0.4647 (5)	0.1399 (3)	0.0748 (14)	
C6	0.4956 (2)	0.5251 (5)	0.3664 (3)	0.0748 (14)	
H6	0.5076	0.4445	0.3727	0.090*	
C15	0.3453 (3)	0.7792 (6)	0.4799 (3)	0.109 (2)	
H15A	0.3401	0.7688	0.5320	0.163*	
H15B	0.3398	0.8628	0.4655	0.163*	
H15C	0.3866	0.7537	0.4775	0.163*	

### Atomic displacement parameters (Å^2^)

**Table:**

	*U*^11^	*U*^22^	*U*^33^	*U*^12^	*U*^13^	*U*^23^
Br2	0.0384 (2)	0.0767 (3)	0.0818 (3)	−0.0098 (2)	0.0069 (2)	−0.0024 (3)
Br1	0.0605 (4)	0.1272 (5)	0.0961 (4)	0.0137 (3)	−0.0018 (3)	0.0346 (4)
O2	0.0579 (18)	0.0531 (17)	0.0452 (16)	−0.0035 (13)	0.0171 (13)	0.0031 (13)
C17	0.038 (2)	0.041 (2)	0.042 (2)	−0.0016 (16)	0.0067 (16)	−0.0005 (17)
C11	0.040 (2)	0.036 (2)	0.044 (2)	−0.0041 (16)	0.0086 (16)	−0.0025 (16)
N1	0.048 (2)	0.0387 (19)	0.064 (2)	0.0000 (15)	0.0124 (16)	−0.0055 (17)
C10	0.045 (2)	0.049 (2)	0.046 (2)	−0.0029 (18)	0.0093 (18)	0.0037 (18)
C12	0.039 (2)	0.036 (2)	0.049 (2)	−0.0044 (16)	0.0121 (17)	−0.0028 (17)
O3	0.105 (3)	0.0497 (19)	0.079 (2)	−0.0167 (18)	0.037 (2)	0.0120 (16)
C13	0.036 (2)	0.047 (2)	0.054 (2)	−0.0041 (17)	0.0122 (18)	0.004 (2)
C9	0.056 (3)	0.048 (2)	0.057 (3)	−0.006 (2)	0.021 (2)	0.006 (2)
C7	0.039 (2)	0.045 (2)	0.052 (2)	−0.0061 (17)	0.0164 (18)	−0.0018 (18)
O1	0.076 (2)	0.087 (2)	0.073 (2)	−0.0251 (19)	0.0141 (17)	0.0293 (19)
C18	0.046 (2)	0.055 (2)	0.049 (2)	−0.0012 (19)	0.0135 (19)	−0.002 (2)
C22	0.046 (2)	0.056 (3)	0.054 (2)	−0.0102 (19)	0.0110 (19)	−0.015 (2)
C4	0.037 (2)	0.050 (2)	0.059 (3)	−0.0004 (18)	0.0198 (19)	0.005 (2)
C5	0.064 (3)	0.055 (3)	0.075 (3)	−0.002 (2)	0.012 (2)	−0.001 (2)
C1	0.038 (2)	0.080 (3)	0.068 (3)	0.002 (2)	0.012 (2)	0.014 (3)
C19	0.046 (2)	0.053 (2)	0.050 (2)	−0.0054 (19)	0.0004 (19)	−0.0075 (19)
C20	0.036 (2)	0.047 (2)	0.057 (3)	−0.0054 (17)	0.0056 (18)	0.0036 (19)
C14	0.070 (3)	0.086 (3)	0.050 (3)	0.005 (3)	0.019 (2)	0.010 (2)
C8	0.044 (2)	0.051 (2)	0.060 (3)	−0.0136 (19)	0.0130 (19)	0.003 (2)
C21	0.044 (2)	0.072 (3)	0.063 (3)	−0.008 (2)	0.021 (2)	−0.010 (2)
C2	0.049 (3)	0.076 (3)	0.078 (3)	−0.005 (2)	−0.004 (2)	−0.007 (3)
C3	0.052 (3)	0.057 (3)	0.079 (3)	0.004 (2)	0.000 (2)	−0.001 (2)
C16	0.079 (4)	0.055 (3)	0.088 (4)	0.002 (3)	0.015 (3)	−0.008 (3)
C6	0.067 (3)	0.062 (3)	0.094 (4)	0.018 (3)	0.015 (3)	0.017 (3)
C15	0.137 (6)	0.129 (5)	0.063 (3)	−0.042 (5)	0.028 (4)	−0.023 (3)

### Geometric parameters (Å, º)

**Table:**

Br2—C20	1.904 (4)	C22—C21	1.381 (5)
Br1—C1	1.896 (4)	C22—H22	0.9300
O2—C13	1.312 (5)	C4—C3	1.373 (6)
O2—C14	1.466 (5)	C4—C5	1.378 (6)
C17—C18	1.378 (5)	C5—C6	1.392 (7)
C17—C22	1.388 (5)	C5—H5	0.9300
C17—C11	1.515 (5)	C1—C2	1.351 (7)
C11—C10	1.530 (5)	C1—C6	1.361 (7)
C11—C12	1.560 (5)	C19—C20	1.365 (5)
C11—H11	0.9800	C19—H19	0.9300
N1—C16	1.150 (5)	C20—C21	1.375 (5)
N1—C12	1.437 (5)	C14—C15	1.451 (7)
C10—C9	1.495 (5)	C14—H14A	0.9700
C10—H10A	0.9700	C14—H14B	0.9700
C10—H10B	0.9700	C8—H8A	0.9700
C12—C13	1.534 (5)	C8—H8B	0.9700
C12—C7	1.585 (5)	C21—H21	0.9300
O3—C13	1.190 (5)	C2—C3	1.380 (6)
C9—O1	1.206 (4)	C2—H2	0.9300
C9—C8	1.507 (6)	C3—H3	0.9300
C7—C4	1.516 (5)	C6—H6	0.9300
C7—C8	1.539 (5)	C15—H15A	0.9600
C7—H7	0.9800	C15—H15B	0.9600
C18—C19	1.389 (5)	C15—H15C	0.9600
C18—H18	0.9300		
			
C13—O2—C14	116.8 (3)	C5—C4—C7	120.6 (4)
C18—C17—C22	117.7 (3)	C4—C5—C6	121.5 (5)
C18—C17—C11	123.3 (3)	C4—C5—H5	119.2
C22—C17—C11	118.9 (3)	C6—C5—H5	119.2
C17—C11—C10	113.7 (3)	C2—C1—C6	120.0 (4)
C17—C11—C12	114.0 (3)	C2—C1—Br1	119.3 (4)
C10—C11—C12	109.5 (3)	C6—C1—Br1	120.6 (4)
C17—C11—H11	106.3	C20—C19—C18	119.4 (4)
C10—C11—H11	106.3	C20—C19—H19	120.3
C12—C11—H11	106.3	C18—C19—H19	120.3
C16—N1—C12	178.1 (4)	C19—C20—C21	121.1 (4)
C9—C10—C11	111.1 (3)	C19—C20—Br2	119.9 (3)
C9—C10—H10A	109.4	C21—C20—Br2	119.0 (3)
C11—C10—H10A	109.4	C15—C14—O2	109.4 (4)
C9—C10—H10B	109.4	C15—C14—H14A	109.8
C11—C10—H10B	109.4	O2—C14—H14A	109.8
H10A—C10—H10B	108.0	C15—C14—H14B	109.8
N1—C12—C13	106.9 (3)	O2—C14—H14B	109.8
N1—C12—C11	109.8 (3)	H14A—C14—H14B	108.2
C13—C12—C11	109.6 (3)	C9—C8—C7	110.5 (3)
N1—C12—C7	107.2 (3)	C9—C8—H8A	109.5
C13—C12—C7	112.7 (3)	C7—C8—H8A	109.5
C11—C12—C7	110.6 (3)	C9—C8—H8B	109.5
O3—C13—O2	126.2 (4)	C7—C8—H8B	109.5
O3—C13—C12	124.2 (4)	H8A—C8—H8B	108.1
O2—C13—C12	109.6 (3)	C20—C21—C22	118.8 (4)
O1—C9—C10	122.5 (4)	C20—C21—H21	120.6
O1—C9—C8	122.4 (4)	C22—C21—H21	120.6
C10—C9—C8	115.0 (3)	C1—C2—C3	120.1 (5)
C4—C7—C8	113.6 (3)	C1—C2—H2	119.9
C4—C7—C12	114.9 (3)	C3—C2—H2	119.9
C8—C7—C12	111.8 (3)	C4—C3—C2	122.0 (4)
C4—C7—H7	105.2	C4—C3—H3	119.0
C8—C7—H7	105.2	C2—C3—H3	119.0
C12—C7—H7	105.2	C1—C6—C5	119.6 (5)
C17—C18—C19	121.3 (4)	C1—C6—H6	120.2
C17—C18—H18	119.4	C5—C6—H6	120.2
C19—C18—H18	119.4	C14—C15—H15A	109.5
C21—C22—C17	121.8 (4)	C14—C15—H15B	109.5
C21—C22—H22	119.1	H15A—C15—H15B	109.5
C17—C22—H22	119.1	C14—C15—H15C	109.5
C3—C4—C5	116.7 (4)	H15A—C15—H15C	109.5
C3—C4—C7	122.6 (4)	H15B—C15—H15C	109.5
			
C18—C17—C11—C10	41.2 (5)	C11—C12—C7—C8	16.0 (4)
C22—C17—C11—C10	−135.7 (4)	C22—C17—C18—C19	1.2 (6)
C18—C17—C11—C12	−85.3 (4)	C11—C17—C18—C19	−175.8 (4)
C22—C17—C11—C12	97.8 (4)	C18—C17—C22—C21	−1.9 (6)
C17—C11—C10—C9	166.2 (3)	C11—C17—C22—C21	175.2 (4)
C12—C11—C10—C9	−65.0 (4)	C8—C7—C4—C3	13.6 (5)
C16—N1—C12—C13	−174 (100)	C12—C7—C4—C3	−116.9 (4)
C16—N1—C12—C11	67 (14)	C8—C7—C4—C5	−163.9 (4)
C16—N1—C12—C7	−53 (14)	C12—C7—C4—C5	65.7 (5)
C17—C11—C12—N1	54.1 (4)	C3—C4—C5—C6	−1.7 (7)
C10—C11—C12—N1	−74.6 (4)	C7—C4—C5—C6	175.9 (4)
C17—C11—C12—C13	−63.0 (4)	C17—C18—C19—C20	0.8 (6)
C10—C11—C12—C13	168.3 (3)	C18—C19—C20—C21	−2.2 (6)
C17—C11—C12—C7	172.1 (3)	C18—C19—C20—Br2	176.7 (3)
C10—C11—C12—C7	43.5 (4)	C13—O2—C14—C15	140.1 (5)
C14—O2—C13—O3	−0.2 (6)	O1—C9—C8—C7	−139.2 (4)
C14—O2—C13—C12	178.9 (3)	C10—C9—C8—C7	39.5 (5)
N1—C12—C13—O3	−2.8 (5)	C4—C7—C8—C9	168.9 (3)
C11—C12—C13—O3	116.1 (4)	C12—C7—C8—C9	−59.1 (4)
C7—C12—C13—O3	−120.3 (4)	C19—C20—C21—C22	1.5 (6)
N1—C12—C13—O2	178.1 (3)	Br2—C20—C21—C22	−177.4 (3)
C11—C12—C13—O2	−62.9 (4)	C17—C22—C21—C20	0.6 (7)
C7—C12—C13—O2	60.6 (4)	C6—C1—C2—C3	−1.7 (8)
C11—C10—C9—O1	−159.9 (4)	Br1—C1—C2—C3	177.9 (4)
C11—C10—C9—C8	21.4 (5)	C5—C4—C3—C2	2.4 (7)
N1—C12—C7—C4	−93.0 (4)	C7—C4—C3—C2	−175.2 (4)
C13—C12—C7—C4	24.3 (4)	C1—C2—C3—C4	−0.7 (8)
C11—C12—C7—C4	147.3 (3)	C2—C1—C6—C5	2.4 (7)
N1—C12—C7—C8	135.7 (3)	Br1—C1—C6—C5	−177.3 (4)
C13—C12—C7—C8	−107.0 (4)	C4—C5—C6—C1	−0.6 (7)

### Hydrogen-bond geometry (Å, º)

**Table:**

*D*—H···*A*	*D*—H	H···*A*	*D*···*A*	*D*—H···*A*
C11—H11···O3^i^	0.98	2.58	3.226 (5)	123

Symmetry code: (i) −*x*+1/2, *y*+1/2, −*z*+1/2.
